# Calcium Phosphate Nanoparticles-Based Systems for RNAi Delivery: Applications in Bone Tissue Regeneration

**DOI:** 10.3390/nano10010146

**Published:** 2020-01-14

**Authors:** Tanya J. Levingstone, Simona Herbaj, John Redmond, Helen O. McCarthy, Nicholas J. Dunne

**Affiliations:** 1 School of Mechanical and Manufacturing Engineering, Dublin City University, 9 Dublin, Ireland; tanya.levingstone@dcu.ie (T.J.L.); simona.herbaj2@mail.dcu.ie (S.H.); john.redmond23@mail.dcu.ie (J.R.); 2 Centre for Medical Engineering Research, School of Mechanical and Manufacturing Engineering, Dublin City University, 9 Dublin, Ireland; 3 Advanced Processing Technology Research Centre, Dublin City University, 9 Dublin, Ireland; 4 Trinity Centre for Biomedical Engineering, Trinity Biomedical Sciences Institute, Trinity College Dublin, 2 Dublin, Ireland; 5 School of Pharmacy, Queen’s University Belfast, Belfast BT9 7BL, UK; h.mccarthy@qub.ac.uk; 6 Department of Mechanical and Manufacturing Engineering, School of Engineering, Trinity College Dublin, 2 Dublin, Ireland; 7 Advanced Materials and Bioengineering Research Centre (AMBER), Royal College of Surgeons in Ireland and Trinity College Dublin, 2 Dublin, Ireland

**Keywords:** bone tissue engineering, calcium phosphates, gene therapy, nanoparticles, non-viral vectors, RNA interference, surface functionalization

## Abstract

Bone-related injury and disease constitute a significant global burden both socially and economically. Current treatments have many limitations and thus the development of new approaches for bone-related conditions is imperative. Gene therapy is an emerging approach for effective bone repair and regeneration, with notable interest in the use of RNA interference (RNAi) systems to regulate gene expression in the bone microenvironment. Calcium phosphate nanoparticles represent promising materials for use as non-viral vectors for gene therapy in bone tissue engineering applications due to their many favorable properties, including biocompatibility, osteoinductivity, osteoconductivity, and strong affinity for binding to nucleic acids. However, low transfection rates present a significant barrier to their clinical use. This article reviews the benefits of calcium phosphate nanoparticles for RNAi delivery and highlights the role of surface functionalization in increasing calcium phosphate nanoparticles stability, improving cellular uptake and increasing transfection efficiency. Currently, the underlying mechanistic principles relating to these systems and their interplay during in vivo bone formation is not wholly understood. Furthermore, the optimal microRNA targets for particular bone tissue regeneration applications are still unclear. Therefore, further research is required in order to achieve the optimal calcium phosphate nanoparticles-based systems for RNAi delivery for bone tissue regeneration.

## 1. Introduction

Despite bone’s intrinsic ability to repair itself without scarring, 5–10% of all bone fractures result in delayed or non-union fractures [1], causing chronic pain for patients. This impacts significantly on patient quality of life and places a significant burden on the health system with the cost of treating non-union fractures in the USA expected to rise from $19 billion (2005) per annum to $25 billion by 2025 [2]. Autologous bone grafts, the “gold standard” currently employed to treat delayed or non-union fractures, exhibit a high incidence of failure and numerous limitations, including donor site morbidity, lack of tissue availability, and invasive surgery [3]. Similar drawbacks, such as unfavorable immune responses, rejection rates and lack of graft availability, are found in the use of allografts and xenografts, whereas synthetic bone graft substitutes often lack biocompatibility and osteogenic potential [4]. The combination of protein therapy with synthetic bone grafts has demonstrated promising early results. However, the low retention rate and high concentration of protein required to obtain a biological effect is a cause for concern [5]. Consequently, there is an increasing need for and interest in the development of new, effective therapies for bone regeneration.

Gene therapy is considered as the latest approach for the repair of challenging bone defects-delivering exogenous deoxyribonucleic acid (DNA) or ribonucleic acid (RNA) to obtain controlled and sustained protein expression at the fracture site [6]. In particular over the last two decades, the important role played by RNA interference (RNAi) mediated gene repression/silencing in bone metabolism through the regulation of the proliferation, differentiation and function of bone cells has been recognized and RNA-based therapies have shown promise for bone regeneration [7]. In contrast to DNA-based technologies, RNA-based approaches offer the advantage of utilizing a cell’s own internal machinery to alter the gene expression. Furthermore, as RNA-based therapeutics do not require nuclear entry, these molecules completely avoid the risk of insertional mutagenesis and therefore present a safer and more viable alternative [8]. However, the delivery of RNA is often hampered by its susceptibility to degradative enzymes, which present a significant limitation to its use. [8,9]. Furthermore, its poor capacity to penetrate the host cell membrane and selectively distribute to the desired tissues or cells within the body presents a significant barrier to clinical translation [7]. Thus, the establishment of a carrier that would increase protection, intracellular release and expression of genetic material is imperative. Calcium phosphate nanoparticles hold particular potential in this regard for bone-related conditions as they have a strong affinity for binding to nucleic acids [10,11]. They are also well-accepted by the body and have a significant surface-to-volume ratio that allows for a higher driving force for diffusion, increased particle solubility and adhesion to specific proteins [12]. Furthermore, calcium phosphate-based systems are osteoconductive, osteoinductive, and the majority are considered bioresorbable [13]. This review provides a synopsis of the current state-of-the-art relating to the design of calcium phosphate nanoparticles as non-viral vectors and their application in RNA-based therapy for bone tissue regeneration. The potential for surface functionalization methods to improve the stability, transfection and safety of calcium phosphate will also be discussed.

## 2. RNA Interference for Bone Repair

### 2.1. Biogenesis of microRNA and siRNA

**Figure 1:**
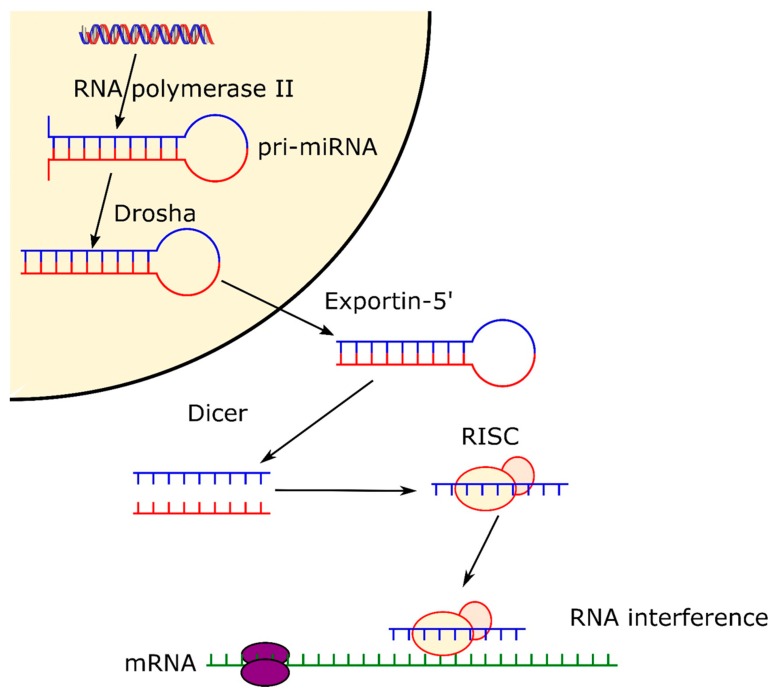
Pathway of microRNA, from formation in the cell nucleus thanks to RNA polymerase II, to formation of RISC complex and expression.

RNAi offers significant prospects for the development of next generation bone tissue engineering therapeutics. Two types of RNA are fundamental to RNAi, microRNA (miRNA) and silencing RNA (siRNA). miRNAs are naturally occurring non-coding single strand hairpin of RNA about 20–25 nucleotides long. Primary miRNAs (pri-miRNA) are produced by RNA polymerase II and appear as a longer sequence of RNA which includes the miRNA hairpin shape. This is recognized by the endonuclease Drosha that proceeds to the cleavage of the hairpin from the pri-miRNA at about one helical turn from the base of the hairpin [14]. Subsequently, the hairpin exits the nucleus thanks to the exportin-5 complex, where it meets another endonuclease, Dicer, which cleaves the top of the hairpin, separating the two strands [14]. One of those strands is degraded whereas the other one is then loaded onto an Argonaut protein, forming the RNA-induced silencing complex (RISC) [15]. Finally, once the miRNA is loaded onto the protein, it can control gene expression by directing the Argonaut protein towards the 3′-untraslated regions (3′-UTRs) of other messenger RNAs (mRNA) [16]. Therefore, miRNAs are directly involved in the regulation of the expression of different genes at post-transcriptional level [17] by binding to reception sites within the cells, and guiding cell proliferation, differentiation, and death (Figure 1). miRNAs can stimulate the expression of proteins such as bone morphogenetic proteins (BMPs) that are intrinsically related to osteogenesis differentiation. miRNAs are also directly involved in pathways leading to different bone conditions such as osteoporosis and osteoarthritis, as they can upregulate or downregulate genes related to those diseases [18].

siRNAs are double stranded RNAs that are widely studied for specific gene silencing. The siRNAs are processed by Dicer, which cleaves them to short sequences of approximately 20 nucleotides and subsequently bind to an Argonaut protein, forming the RNA-induced silencing complex (RISC), similarly to the process followed by miRNAs [19]. Once part of the RISC, one strand of the siRNA is degraded, while the other strand, called the “guide strand” remains bound to the Argonaut protein. The siRNAs are able to perfectly match specific sites onto the mRNA, therefore silencing the expression of specific genes by endo nucleolytic cleavage of the mRNA [19], “knocking-down” the targeted gene. However, despite the high specificity of siRNA, sometimes their use can trigger an “off-target” gene [19]. This happens when the structure of the off-target gene is similar to the one of the intended target. There are a number of strategies available in order to overcome this problem: benefitting from siRNA redundancy; applying chemical modification of the siRNA; or eliminating pro-inflammatory sequences to avoid immune-stimulation caused by the siRNA [20]. A more in-depth analysis of the biogenesis of miRNA and siRNA along with the mechanisms of action has been reviewed elsewhere [14,15,16].

### 2.2. Bone Interfering miRNA

**Figure 2:**
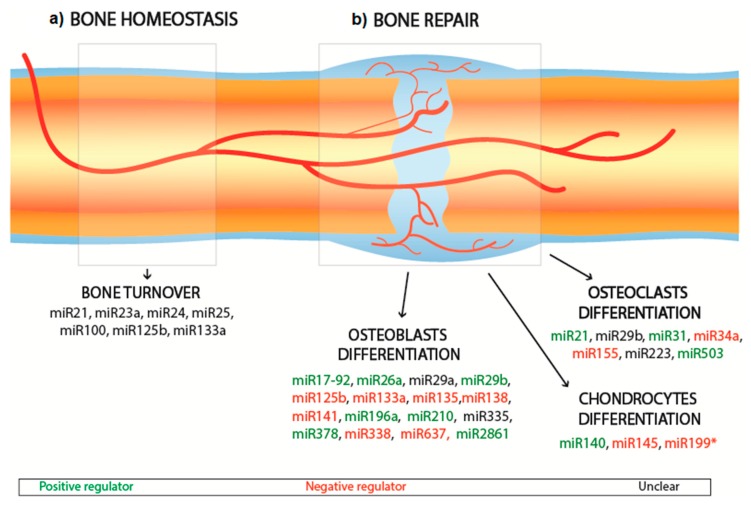
Schematic of miRNAs involved in bone homeostasis (**a**) and bone repair (**b**). The miRNAs involved in bone repair are distinguished in positive regulators (green), negative regulators (red), unclear (black).

MiRNAs are shown to regulate the crosstalk between transcription factors, including osteoblast differentiation, osteoclasts and osteoblasts activation, bone remodeling, and bone diseases metabolism. Within the large spectra of miRNAs involved in the regulation of osteogenesis promotion and/or inhibition, some miRNAs have been shown to remarkably affect the bone regulating process. To date, a relatively low number of miRNAs have been investigated, leaving significant scope for further development. The effects of the principal bone interfering miRNAs which have been shown to directly interfere with the differentiation pathways of bone cells are summarized in Figure 2 and Table 1. Figure 2 highlights the miRNAs that upregulate or downregulate the differentiation of osteoblasts, osteoclasts and chondrocytes during bone repair, as well as the miRNAs that are believed to affect bone homeostasis overall, by balancing bone turnover. Of the miRNAs that influence osteoblasts differentiation, miR-26a has been most extensively investigated to date. Delivery of miR-26a has been shown to increase the expression of bone-related factors (e.g., Runt-related transcription factor 2 (RUNX2), osteocalcin (OCN) and bone morphogenetic protein-2 (BMP-2)), and vascular-related factors (e.g., VEGF and Ang1) [21] and to target the glycogen synthase kinase 3-beta (GSK-3β) gene, which is responsible for augmenting bone mineralization and bone mass [22,23]. Similarly, miR-148b has been identified as a potent bone promoter [24], acting to downregulate the noggin expression, which works as an antagonist of BMPs and is produced by the NOG gene [25]. Liao et al. showed that the co-expression of miR-148b and BMP-2 can lead to an enhancement in osteogenesis compared to the separate delivery of miR-148b and BMP-2 [26].

Current RNAi studies tend to focus on RUNX2 as a critical target for miRNAs since it represents a master transcription factor and is heavily involved in the regulation of multiple genes promoting osteogenesis, extracellular matrix (ECM) synthesis and bone formation [61]. Indeed, RUNX2 inhibition has severe consequences for the ability of cells to differentiate into osteoblasts [62]. RUNX2 can also promote and guide the expression of bone regulating factors such as OCN, osteopontin (OPN), and alkaline phosphatase (ALP). To enhance osteogenesis, it is possible to fabricate miRNAs to antagonize (known as antagomiRNAs) the action of miRNAs such as miR-133 to ultimately promote the expression of RUNX2 [32,33]. This approach was successfully implemented by Mencía Castaño et al. with the incorporation of antagomiR-133a into hydroxyapatite (HA) nanoparticles loaded onto a collagen scaffold [33]. These complexes inhibited miR-133 activity in human mesenchymal cells culture with minimal cytotoxicity. Similarly, miR-135 prevents osteogenesis by negatively interfering with Smad 5, a transducer of BMP, hence disrupting the osteogenic differentiation pathway [32]. Overexpression of miR-206 is related to osteogenic inhibition by acting on Connexin-43-a critical gap junction for osteoblast communication [63]. Finally, miR125b is found to inhibit osteoblast differentiation by acting on Osterix, a transcription factor critical for the regulation of the bone formation and bone remodeling. Indeed, Osterix-deficient mice do not survive after birth due to the incomplete formation of the skeletal system [64], whereas Osterix-deactivated mice show spinal deformities [65]. However, for some miRNAs, the impact on osteoblasts remains unclear. For example, the overexpression of miR-335 has been shown to lead to inhibition of human mesenchymal stem cells (hMSCs) proliferation and osteogenic potential [42]; whereas other studies have reported that miR-335-5p acts upon the Wnt signaling pathways that promote osteogenesis [41].

While much of the current research is focused on enhancing osteoblast differentiation, miRNAs that affect osteoclast differentiation have also been investigated. For example, miR31 has been shown to enhance osteoclastogenesis by targeting RhoA under RANKL stimulation [52]; and miR-21 can induce osteoclastogenesis by downregulating the levels of programmed cell death 4 (PDCD4) protein [50]. miR-21 has also been shown to positively correlate with risk of osteoporosis [66]. Conversely, some miRNAs have been shown to inhibit osteoclastogenesis, specifically miR-503, miR-155 and miR-34a, through targeting some osteoclasts regulator factors [53,54,57]. Similarly, as detailed in Figure 2 and Table 1, miRNAs (e.g., miR-140) that upregulate chondrocyte differentiation [58] and others (e.g., miR-199) that downregulate it [60] by interfering with the differentiation pathway have been identified. Alternatively, bone repair can be addressed by targeting miRNAs that are believed to play an essential role in regulating the healthy turnover of bone cells, also known as bone homeostasis. The discovery of most miRNAs is related to the study of osteoporosis, as this disease is intrinsically related to an imbalance between bone resorption and bone formation. Therefore, the study of these miRNA (e.g., miR-21, miR-23a, miR-24, miR-25 and miR-100) is thought to provide useful insights on the regulation of bone homeostasis [56], and could potentially be applied to achieve bone repair.

## 3. Mechanisms of miRNA Delivery for Bone Repair

Efficient, sustained and safe delivery of genetic material is essential for successful RNAi mediated gene repression, regulation and silencing. The promise of therapeutic benefits from distribution of exogenous DNA/RNA is currently limited by the lack of effective delivery systems. Current techniques for gene delivery include physical methods [67] and vector-based systems [68,69]. Physical methods for nucleic acid delivery include: naked DNA/RNA injection/microinjection [70], use of biolistic/gene gun [68], electroporation [71], and sonoporation [69]. Despite successful applications of these physical delivery techniques, significant hurdles exist impeding their use for gene therapies. In particular, genetic material delivered using these techniques rapidly comes under attack from endonucleases resulting in a poor half-life of DNA/RNA, leading to a low likelihood of the gene therapy reaching its destination–first to the cell and then the nucleus. The incorporation and/or encapsulation of genetic cargo using vectors/carriers has resulted in greater success. These vector-based systems can be sub-divided into two further classes: (1) Viral [72] and (2) non-viral [73] systems. Viral and non-viral approaches used in RNA-based therapies have reviewed extensively within the literature [7,74,75].

### 3.1. Viral Vectors

Viral vectors are considered to be highly effective methods of nucleic acid delivery as they provide high transfection rates taking advantage of the intrinsic mechanisms viruses have to infect host cells [76]. Use of viral vectors (e.g., adenoviruses, retroviruses, adeno-associated viruses and lentiviruses) for therapeutic delivery applications [76,77,78,79] has demonstrated the successful intracellular delivery of pDNA for generation of RNAi molecules including for bone-related conditions [80,81,82]. However, some limitations and safety concerns related to using viral vectors has resulted in a low number of clinical products being approved by the Food and Drug Administration (FDA), despite their excellent efficiency of transfection. Insertional mutagenesis poses a significant risk with use of viral vectors that involve the integration of their nucleic cargo to the host genome. Risk of carcinogenesis is also an important concern with regards to insertional mutagenesis, albeit a low risk, whereby proto-oncogenes or genes involved in cell cycle regulation are affected resulting in tumor formation [83,84,85,86]. Immunogenicity also poses a significant barrier because viral vectors have the potential to provoke a robust immune response post administration in vivo, resulting in the need for vector modification to overcome such complications [86]. Additionally, the general lack of cost-effectiveness and the challenging nature of vector production and scale-up [87,88] has seen a seismic shift in research focus to transgene delivery using safer alternatives (e.g., non-viral vectors).

### 3.2. Non-Viral Vectors

The use of non-viral vectors for the DNA delivery for bone repair is well established and more recently these approaches are being explored for the delivery of RNA agents [89]. Non-viral vectors composed of organic or inorganic-based materials have been used successful in RNA-based therapies. Polyethylenimine (PEI) have been widely studied as non-viral vectors for gene delivery, however high molecular weight PEI is highly toxic to cells and low molecular weight PEI has low transfection efficiencies [22]. As a result, PEI has been combined with other polymers offering improved biocompatibility. Specific combinations include the following PEI/poly(lactic-co-glycolic acid) (PLGA) [90] and PEI/poly(ethylene)glycol (PEG) [22]. Chitosan [91]) and cationic lipids (e.g., Lipofectamine^®^2000 [22,92], aptamer-functionalized lipids [93] and 1,2-Dioleyl-3-trimethylammonium-propane (DOPA) [94]) have also shown promise in this regard [95]. Inorganic vectors include calcium phosphate (CaP) [96,97], bioactive glass [98,99], carbon nanotubes [100], silica [101], and gold [102]. Non-viral vectors confer a range of advantages over their viral counterparts: they can deliver larger genetic cargos, have reduced immunogenicity/toxicity and are easier to construct and modify [103]. The application of non-viral vectors also allows delivery of synthetic siRNA/miRNA mimics, thereby avoiding the need for nuclear localization as per pDNA constructs containing RNAi expression cassettes. This action enables the direct interaction of siRNA/miRNA with RNAi machinery in the cytosol, reducing the extent of the intracellular trafficking required for RNAi mediated gene repression and silencing. However, despite these benefits, a significant barrier exists between non-viral vectors and their approved clinical usage due primarily to their poor transfection efficiency [103]. Other concerns relate to undesirable off-target or on-target effects, short half-life under physiological conditions and the potential for an unfavorable immune response. This review focusses specifically on the use of calcium phosphate nanoparticles for RNA delivery for bone-related conditions, and the various surface modifications and functionalization strategies that can be employed to ensure effective targeted delivery.

## 4. Calcium Phosphates Nanoparticles as Non-Viral Vectors

The natural occurrence of calcium phosphate within the body, specifically in bone and tooth enamel, makes them a logical choice for RNA delivery for bone repair applications [2]. Ca^2+^ and ${\mathrm{PO}}_{4}^{3-}$ ions play a critical role in the regulation of bone resorption and bone deposition [104]. In particular, Ca^2+^ is shown to actively induce chemotaxis, attracting cells such as monocytes, osteoblasts, and hematopoietic stem cells to the site of injury [105]. Ca^2+^ is also shown to induce proliferation and osteoblast differentiation [106], along with the expression of osteogenic genes [107]. Similarly, ${\mathrm{PO}}_{4}^{3-}$ is involved in osteoblast proliferation and differentiation [108] via entering the mitochondria and stimulating the production of adenosine triphosphate (ATP), which converts to adenosine and promotes osteogenesis [109,110]. Furthermore, calcium phosphate nanoparticles have specific properties that make them attractive as delivery vectors for RNA. Calcium phosphate has a high binding affinity to various molecules including RNA, with binding occurring through electrostatic interaction between Ca^2+^ in the CaP carrier and phosphate groups in the RNA structure [2]. Calcium phosphate is easily endocytosed by cells through the lipid bilayer cellular membrane and dissolves within the acidic environment in endosomes and lysosomes leading to the release of nucleic acid in the targeted region within the cell [111]. Calcium phosphate nanoparticles are biocompatible, biodegradable, non-toxic and non-immunogenic [111]. Additionally, calcium phosphate nanoparticles have demonstrated improved cytocompatibility compared to Lipofectamine^TM^2000 thus suggesting a better applicability in vivo [112]. Calcium phosphate nanoparticles also demonstrate osteogenic properties that make them particularly attractive as non-viral vectors for bone tissue engineering applications [113,114]. The use of calcium phosphate nanoparticles as non-viral delivery vectors has also been shown to promote enhanced osteogenic differentiation of bone marrow-derived MSCs compared to PEI [115]. Furthermore, calcium phosphate-based powders are inexpensive and easily synthesized nanocarriers and a high level of safety relating to the use of calcium phosphate nanoparticles for cell transfection has been reported [74,75,116].

The use of calcium phosphate for gene delivery was first demonstrated by Graham and Van der Eb [117]. They realized that producing calcium phosphate in a DNA rich aqueous solution would lead to the spontaneous formation of nano-sized DNA loaded calcium phosphate without interfering with the calcium phosphate structure [117]. Since the application of high energy methods, such as the use of high temperature or high shear stress, has the potential to degrade the genetic cargo quickly, the main route for synthesizing calcium phosphate nanoparticles for gene delivery is the wet co-precipitation method [118]. Control over the main reaction parameters (e.g., temperature, pH, reaction time and precursor concentrations) is important to enable optimization of the particle properties for gene delivery applications and to ensure reproducibility [11]. Welzel et al. reported the use of a controlled wet-precipitation method for the synthesis of spherical DNA loaded calcium phosphate nanoparticles with a mean particle size of 10–20 nm [119]. A similar methodology was used by Mencía Castaño et al. to fabricate nHA particles complexed with both miR-mimics and antagomiRs forming nanomiRs [33,96,97].

The family of calcium phosphate-base materials is commonly characterized based upon chemical composition, crystallinity, and morphology [120]. The solubility of calcium phosphates is determined by their Ca/P ratio, crystallinity, phase purity and the pH of the local environment, with pure crystalline HA exhibiting the highest Ca/P ratio and least solubility in a physiological environment leading to slower resorption kinetics in vivo [2,120,121]. To be successful as a vector for RNA delivery it is necessary for calcium phosphate particles to remain stable within the hostile extracellular environment in order to protect the molecular cargo. Once inside the cell, transfection efficiency is dependent on the ability of RNA to escape from the endosome. Since endosomal escape is directed by the dissolution behavior of the carrier, faster dissolution leads to a faster increase in osmotic pressure and thus earlier endosome escape [122]. Calcium phosphate nanoparticles thus have particular advantages for RNA delivery since nanoparticle dissolution can be triggered by the acidic environment found inside the cell and thus extracellular liberation of the delivery cargo can be prevented. The release of the genetic cargo from endocytosed calcium phosphate nanoparticles relies on the ability of calcium phosphate to dissolve efficiently in the acidic environment within the endosome [123]. Ruvinov et al. demonstrated that the pH dependent reversibility of the RNA-calcium phosphate complex can be exploited for beneficial endosomal escape [124]. They proposed that the local environment with high calcium concentration formed inside the endosome due to the uptake of calcium–siRNA complexes, could lead to highly increased proton influx into the endosome. This will then lead to passive entrance of chloride ions and water molecules causing osmotic swelling, endosomal membrane rupture and escape of its contents. This calcium efflux could promote the destabilization and decomplexation of calcium–siRNA structures, leaving the siRNA to act freely in the cytoplasm upon endosomal rupture. The excess of calcium ions inside the cell could then be promptly removed by calcium pumps on the cell membrane [124]. Goldshtein et al., have also confirmed that Ca^2+^ ions detach from the siRNA once in the endosomal acidic environment, escaping from the endosome and allowing protons to enter, leading to swelling and eventual rupture of the early endosome [125].

While dissolution of calcium phosphate nanoparticles is essential in order to achieve release of the genetic cargo, high intracellular Ca^2+^ levels released during calcium phosphate dissolution can lead to cell death [2]. Thus consideration of both the size and solubility of calcium phosphate nanoparticles is important in order to minimize cytotoxic effects on cells [121,126]. While HA remains the most frequently used calcium phosphate in gene delivery applications to date, amorphous calcium phosphate (ACP), beta tricalcium phosphate and dicalcium phosphate dihydrate (DCPD) have also shown promise [122,127]. There is significant scope for investigation of other calcium phosphate phases and the development of biphasic calcium phosphate-based materials that are specifically designed with tailored resorption kinetics for effective delivery of RNA [128]. Additionally, the substitution of ions such as Mg^2+^, CO_2_^3^, K^+^, Sr and Al^3+^, into the calcium phosphate crystal lattice has been shown to result in enhanced cellular uptake and thus may lead to increased efficiencies of RNA delivery [111,129,130,131].

The mean particle size, shape and charge are pivotal parameters for ensuring that calcium phosphate nanoparticles loaded with genetic material can be internalized by cells. Adair et al. report that intracellular internalization requires positively charged particles of less than 200 nm in size [132]. Olton et al. report the ideal calcium phosphate particle size for non-viral gene delivery to be between 25 and 50 nm [133]. Dorozhkin reported similar results demonstrating that calcium phosphate nanoparticles exhibiting a mean particle size of 20 ± 5 nm result in the most significant cellular uptake by osteoblast-like cell lines [12]. Regarding shape-porous, sphere-like nanoparticles are recognized as being preferable not only for gene delivery but also for cell targeting and drug loading [134]. Studies have shown that spherical calcium phosphate nanoparticles can increase osteoblastic proliferation [126] and osteogenic gene expression [135], and limit cell apoptosis [136]. The sizeable surface-area-to-volume ratio offered by calcium phosphate nanoparticles can be exploited to maximise drug loading for therapeutic applications [137]. Recently, mesoporous calcium phosphate nanoparticles (2–50 nm) have received significant attention because of their increased surface area which could potentially be exploited to enhance drug delivery and RNAi encapsulation [138].

The zeta potential or surface charge of calcium phosphate nanoparticles is another crucial parameter for nanoparticle gene delivery as it determines whether the nanoparticle can pass through the cell membrane. Positively charged nanoparticles reportedly result in a higher cellular uptake [139]. However, it is difficult to compare the effect of zeta potential across different studies, as the surface charge is highly sensitive to various sintering routes, different functionalization and experimental conditions [139]. In order to investigate the influence of nanoparticles with differing zeta potential while maintaining the same size and shape, Chen et al. studied the cellular uptake of three compositions of HA nanoparticles that were functionalized with carboxylic acids with the variant of one different functional group [140]. Their work confirmed that positively charged HA nanoparticles are more biocompatible and exhibit a more sustained cellular uptake [140]. Furthermore, the electrostatic interaction between surface charge and adsorbed proteins is dependent on the zeta potential of the calcium phosphate nanoparticles, which is influenced by pH, ionic strength and Ca^2+^ and PO_4_^3−^ ion concentrations [141].

Despite the advantages of calcium phosphate for RNAi delivery, challenges remain. Firstly, the stability of calcium phosphate nanoparticles is limited due to their crystal growth over time [2]. A number of approaches have been investigated to overcome this. Mencía Castaño et al. used surfactant stabilized non-aggregating HA nanoparticles to deliver miR-mimics and antagomiRs [96]. They report internalization efficiencies of 17.4 and 36.5% for miR-mimics and antagomiRs, respectively, with both yielding sustained interfering activity of greater than 90% in monolayer over seven days [96]. This approach was further applied in the delivery of miR-133a inhibiting complexes [33] and antagomiR-16 [97] to enhance human stem cell-mediated osteogenesis. An alternative approach involves the substitution of magnesium (Mg^+^) into the calcium phosphate structure during the co-precipitating reaction leading to a distortion of the atomic structure that inhibits particle growth [142]. Chowdhury report a ten-fold increase in transfection following the incorporation of Mg^+^ into calcium phosphate particles when compared to calcium phosphate alone [129]. However, contradictory results were reported by Goldshtein et al., who suggested that the higher intracellular concentration of Mg^2+^ would impede the cellular uptake of Mg^2+^ doped calcium phosphate [125].

The protection of the genetic cargo from nuclease disruption is a further essential consideration that impacts on the transfection efficiency when using calcium phosphate nanoparticles for gene delivery. Different approaches have been investigated in order to further improve the protection of the genetic cargo and control its rate of release, including the incorporation of genetic material within the core of the calcium phosphate nanoparticle [143] or the use of multi-shell calcium phosphate nanoparticles [2]. The latter configuration provides a much slower degradation profile and further preservation of the genetic cargo [2]. Sokolova et al. successfully developed an approach, which involved preparing a double layer coating of calcium phosphate nanoparticles and DNA, showing an increased transfection efficiency compared to a single layer coating [144]. Multi-shell nanoparticles also allow for a more sustained controlled release and longer storage time of 2–3 months [145]. Tenkumo et al. fabricated multi-shell nanoparticles, by combining HA with functionalized pDNA, which was subsequently loaded in a collagen scaffold for periodontal tissue repair. These functionalized collagen scaffolds exhibited a higher transfection efficacy compared to Lipofectamine^®^2000, a widely used commercial available transfection agent [146].

Overall, the goal for successful delivery of RNA using calcium phosphate nanoparticles is to ensure that: (i) the nanoparticles adhere to the cell membrane without being washed away, (ii) once adhered, the nanoparticles and RNA cargo remain stable for a sufficient amount of time to enable them to be endocytosed by the targeted cells, and (iii) the nanoparticles can escape from the endosome into the cytoplasm in order to activate the RNAi response. Even though the use of calcium phosphate nanoparticles for RNAi delivery has been shown to promote bone repair efficiently, transfection efficiency remains low in comparison to that achieved using viral vectors. Therefore, significant research has been conducted to investigate the influence of surface functionalization of calcium phosphate to facilitate endocytosis of calcium phosphate-based carriers and the consequent endosomal escape [147].

## 5. Functionalized Calcium Phosphate Nanoparticles for Delivery of miRNAs

**Figure 3:**
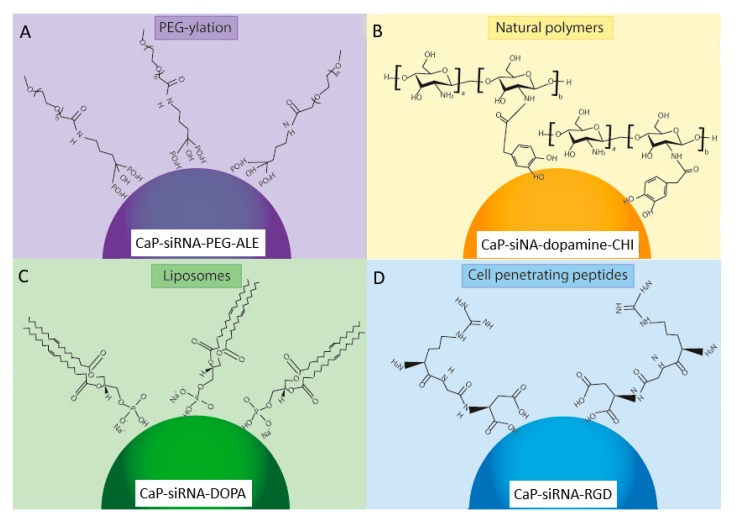
The main methods for surface functionalization of CaP nanoparticles loaded with genetic material (RNAi): (**A**) PEG-ylation, (**B**) Natural polymers, (**C**) Liposomes, (**D**) Cell penetrating peptides. One example of each surface functionalization method is shown. PEG-ylation using PEG and alendronate (PEG-ALE) is shown in (**A**) [155], the natural polymers chitosan and dopamine (-CHI) are shown in (**B**) [156], the use of the liposome 1,2-Dioleoyl-sn-glycero-3-phosphate (DOPA) lipid is shown in (**C**) [157], and the cell penetrating peptide Arginine-Glycine-Aspartic acid (RGD) is shown in (**D**) [74].

The functionalization of calcium phosphate nanoparticles, through the use of PEG-ylation [148], cationic polymer [149], natural polymers [150], biodegradable lipids [151] and cell-penetrating peptides [152] (Figure 3), has been shown to enhance cellular uptake and improve transfection efficiency in gene delivery for a range of applications [111,118,153,154]. While calcium phosphate functionalization methods have not been widely used in date in RNA-based therapies for bone regeneration, they hold significant promise in this regard. The advantages and limitations of these surface functionalization methods are outlined in Table 2.

### 5.1. PEG-Ylation

Poly-ethylene-glycol (PEG) is a hydrophilic polymer that prevents nanoparticle agglomeration by inhibiting particle growth and increases calcium phosphate biocompatibility by decreasing non-specific protein absorption in vivo, which would degrade the RNAi cargo [166]. The use of a PEG shell also helps to minimize the calcium ion release from calcium phosphate following enzymatic degradation in the cytosol, further increasing the biocompatibility of calcium phosphate nanoparticles [111]. Zhang et al. suggested that the thickness of the PEG shell can affect cellular uptake, and proposed an optimal thickness of 10 nm [160]. PEG can be used in conjunction with chelators to further improve its efficacy in stabilizing calcium phosphate nanoparticles, thereby increasing its biocompatibility. Bisphosphonates present a strong affinity for the HA embedded within the bone mineral phase, which confers them with high bone tissue specificity [167]. Therefore, bisphosphonates are widely used in the treatment of various bone diseases (e.g., osteoporosis, Paget’s disease and osteosarcoma). Giger et al. successfully synthesized PEG-bisphosphonate-DNA-calcium phosphate nanoparticles demonstrating a mean particle size of 200 nm, zeta potential of −20 mV, and polydispersity index (PDI) of 0.2–0.3 [168]. These PEG-bisphosphonate-DNA-CaP nanoparticles were stable over 72 h, proving the positive effect of surface functionalization in preventing particle agglomeration. Giger et al. reported ≥60% transfection efficacy comparable to Lipofectamine^®^2000, while maintaining cell viability of about 90% at bisphosphonate concentrations lower than 10 μM, thereby alleviating concerns associated with cytotoxicity of bisphosphonate nanoparticles at low concentration levels [168]. Giger et al. also investigated the use of alendronate (ALE) bound to PEG, which formed a PEG-ALE complex (Figure 3A). The calcium phosphate-siRNA nanoparticles maintained a similar mean particle size (i.e., 260 nm), zeta potential of −17 mV and were stable for over one month, which facilitated good transfection efficacy via clathrin-dependent endocytosis [155]. They also suggested that acidification of the endosome is needed for RNAi cargo release, and highlighted that pH control is crucial for endosomal escape.

pH-responsive calcium phosphate nanoparticles containing a disulphide bond between PEG and siRNA were developed by Zhang et al. [160]. These nanoparticles demonstrated instability when inserted into a reducing environment such as cytosol, allowing for effective cargo release. The use of a disulphide bond also enhanced siRNA uptake by avoiding disruptive interaction between PEG and siRNA [160]. The resultant nanoparticles exhibited a mean particle size of 100 nm and a PDI ≤0.1, confirming the ability of PEG to inhibit calcium phosphate particle growth [160]. Charge conversional polymers pass from anionic to cationic in the presence of low pH, hence facilitating the endosomal escape of the RNAi cargo. Pittella et al. achieved a rapid siRNA release by grafting 1,2-diaminoethane side chain poly{N-[N’-(2-aminoethyl)-2-aminoethyl] aspartamide}, also known as PAsp(DET), with PEG-siRNA-CaP. PAsp(DET) is a good candidate for pH-responsive calcium phosphate nanoparticles because of the protonation behavior of its side chain, which leads to pH-selective membrane destabilization and its biodegradability in physiological conditions [158]. This PAsp(DET)-PEG-siRNA-calcium phosphate approach showed promising results in vivo [169]. Similarly, Lee et al. used citraconic anhydride to graft on PEGylated calcium phosphate to obtain a pH-sensitive surface functionalization of calcium phosphate nanoparticles [170]. Further research in this area has focused on calcium phosphate functionalization using a PEG and poly-aspartic acid (PEG-PAA) copolymer [159]. The resultant calcium phosphate nanoparticles exhibited a mean particle size of 140 nm, and the particle size was reported to be inversely proportional to the amount of PEG-PAA used [171,172]. Kakizawa et al. reported that functionalizing the calcium phosphate-siRNA nanoparticles with PEG-PAA obtained a significant improvement in transfection efficacy showing up to 60% silencing, which confirmed the successful release of siRNA into the cytoplasm [159]. However, the use of PEG-ylation has often raised challenges regarding cellular uptake and endosomal escape, known as ‘PEG dilemma’ [173]. Several approaches have been investigated to overcome this hurdle, including adding specific ligands to target the desired cells, introducing cleavable PEG systems, and investigating ways to readily disrupt the endosomal barrier to free the cargo [173].

### 5.2. Cationic Polymers

Polyethylenimine (PEI) is a hydrophilic polymer, widely used both on its own and in conjunction with other materials as a non-viral vector for cell transfection due to its favorable chemistry. PEI is primarily composed of 25% of primary amines, 50% secondary amines and 25% tertiary amines. Of these amines, the great majority are protonated under in vivo conditions [166]. The non-protonated amines act as a buffer over a wide range of pH and allow for endosomal escape of the genetic cargo. Due to its high amine density, once endocytosed, PEI-encapsulated materials require ATPase enzyme to transport a high number of protons to reach the desired acidification of the endosome, causing an increase in osmotic swelling and subsequent rupture of the endosome; thereby releasing the PEI-encapsulated material [154]. As such, PEI has been used for surface functionalization of calcium phosphate nanoparticles as an outer layer, even though calcium phosphate functionalized with untreated PEI tends to be unstable [161]. This instability causes the formation of nanoparticle agglomerates once in a cellular environment [161]. Neuhaus et al. prepared triple shell calcium phosphate-siRNA-CaP-PEI to increase calcium phosphate stabilization and transfection efficacy, where the outer PEI shell provided a positive charge and calcium phosphate colloidal stability [149]. The nanoparticles demonstrated a mean particle size ≤ 100 nm, a PDI of 0.2–0.3, and zeta potential of 16 mV. The measured transfection efficacy was comparable to Lipofectamine^®^2000, and cell viability remained ≥80%, which demonstrated the calcium phosphate-siRNA-CaP-PEI nanoparticles were moderately cytotoxicity [149]. Devarasu et al. prepared calcium phosphate-siRNA coated nanoparticles using tyrosine-grafted PEI (PEIY) [161]. These calcium phosphate-siRNA nanoparticles demonstrated a mean particle size of 60 nm, a zeta potential of 24–30 mV, and were stable for up to six days [161]. The in vitro transfection showed a silencing efficacy of 95%, and galactose moiety addition to the PEIY coating enhanced the interactions with galactose receptors on hepatocyte and Kupffer cells within the liver leading to improved particle biodistribution in vivo, without affecting the size, stability and in vitro efficiency of the calcium phosphate-siRNA nanoparticles [161].

However, PEI usage is limited because of cytotoxic concerns due to its high charge density. Linear PEI shows higher transfection efficiency and lower cytotoxicity compared to branched PEI and is therefore the preferred option for in vivo applications even though it exhibits lesser amine binding sites [166]. Consequently, PEI modification with PEG grafting is the most common approach to overcome issues associated with cytotoxicity. Petersen et al. reported the degree of substitution for PEI-PEG polymers strongly decreased PEI cytotoxicity and was independent of PEG molecular weight [174]. However, the molecular weight of PEG affected the ability of PEG-PEI copolymers to condense the genetic cargo. PEG with a molecular weight of 5 kDa resulted in the formation of spherical nanoparticles demonstrating a mean particle size of 60 nm, whereas a lower molecular weight PEG (550 Da) resulted in a significant increase in mean particle size of 130 nm [174].

Another cationic polymer used for the surface functionalization of calcium phosphate to enhance cargo release is poly(lactic-co-glycolic acid) (PLGA), which is composed of lactic acid and glycolic acid connected by ester bonding [175]. Wang et al. reported the functionalization of calcium phosphate-pDNA using PLGA-Mpeg for delivery of sizeable genetic cargo. The resultant nanoparticles exhibited a particle size ranging from 20–60 nm and remained highly stable in aqueous solution [176]. Similarly, Tang et al. produced calcium phosphate-pDNA-PLGA nanoparticles that demonstrated a mean particle size of 200 nm and the encapsulation of 95% of pDNA [175]. The transfection efficacy with this approach was relatively low (22%); however, it was significantly improved when compared to that of PLGA-pDNA or calcium phosphate-pDNA nanoparticles [175].

The potential of a triple shell calcium phosphate/siRNA/calcium phosphate/PEI for siRNA delivery for knocking down tumor necrosis factor alpha (TNF-α) in the treatment of tumor cells was investigated by Neuhaus et al. [149]. They successfully reduced gene expression to 18% of the original value, while cell viability remained ≥70% [149]. Zhang et al. also investigated the synthesis of triple shell calcium phosphate nanoparticles in conjunction with siRNA and poly-(l-lysine) embedded in a multi-layered electrolyte film and achieved strong silencing of the OCN and OPN gene expressions on human osteoblasts thus the ability to control bone formation using these novel nanoparticles was confirmed and their potential for use in bone tissue engineering applications demonstrated [177].

### 5.3. Natural Polymers

Natural polymers, including chitosan [162,178] and hyaluronic acid [150,163], have also been used for the surface functionalization of calcium phosphate nanoparticles for gene therapy in bone tissue engineering applications. Chitosan is a natural polysaccharide that offers excellent biocompatibility and biodegradability [162]. It contains many carboxyl groups that can effectively control the synthesis of calcium phosphate nanoparticles by absorbing Ca^2+^ ions and forming chitosan/CaP nanoparticles [114]. It has a high positive charge that leads to enhanced electrostatic interactions between the primary amines within chitosan structure and the negatively charged RNA, thereby protecting RNA during cellular uptake and leading to an increase in transfection efficiency [162]. The transfection efficacy of chitosan is related to its molecular weight, the degree of deacetylation and ratio of nitrogen atoms binding to the phosphate groups of the calcium phosphate [166]. The use of chitosan-glutamine is an exciting approach to enhance calcium phosphate transfection, where high cationic glutamine is used to increase the particle charge of the calcium phosphate nanoparticles. Choi et al. confirmed stabilization of calcium phosphate-siRNA and calcium phosphate-siRNA-chitosan-glutamine nanoparticles and reported a mean particle size reduction from 443 nm to 119 nm and a decrease in PDI from 0.863 to 0.216 [162]. They subsequently loaded a siRNA targeting Noggin into the calcium phosphate nanoparticles that were delivered using a chitosan hydrogel and evaluated transfection efficacy and ALP expression in adipose tissue derived stem cells (ADSCs). Results indicated that the transfection values were comparable to Lipofectamine^®^2000, and ALP production was increased [162].

Surface functionalized of calcium phosphate nanoparticles with chitosan has been shown by Lee et al. to increase serum stability of calcium phosphate-pDNA nanoparticles from 4 h to 24 h (Figure 3B) [156]. Lee et al. further functionalized these nanoparticles for DNA and siRNA delivery by adding dopamine, achieving enhanced nanoparticle stability and significantly reducing the mean particle size to 130 nm [150]. The catechol group of the dopamine molecule was crucial to particle stabilization, acting as a bridge between the calcium ions of calcium phosphate and chitosan. The chitosan-dopamine-siRNA-calcium phosphate nanoparticles displayed enhanced target-gene silencing by siRNA when compared to the chitosan-siRNA-calcium phosphate nanoparticles [156]. Dopamine has also been used with hyaluronic acid; Lee et al. demonstrated that increasing DOPA-hyaluronic acid functionalization resulted in increased particle stability and improved protection of siRNA from enzyme-mediated digestion [150]. A further study by this group demonstrated improved osteogenic differentiation of human bone marrow-derived MSCs (hMSCs) resulting from the co-delivery of a plasmid DNA encoding bone morphogenetic protein 2 and micro RNA 148b using DOPA-hyaluronic acid/CaP [163]. They demonstrated that hyaluronic acid restricted undesirable aggregation and excessive crystal growth of the CaP particles and showed that DOPA-hyaluronic acid/CaP achieved significantly higher transfection in hMSCs than branched polyethylenimine (bPEI, MW 25 kDa) with no cytotoxicity, as a result of the specific interactions between hyaluronic acid and CD44 of bone marrow-derived hMSCs [163].

Finally, an interesting approach is calcium phosphate nanoparticle functionalization with cell targeting molecules. Kozlova et al. reported the surface modification of calcium phosphate nanoparticles for antibody targeting [179]. They prepared calcium phosphate-PEI-siRNA nanoparticles and wrapped them in a silica shell, which was further functionalized by salinization. This functionalization process introduced an amine or thiol group for antibody binding and cellular uptake was confirmed using a human osteoblast-like cell line, MG-63, demonstrating the potential of these particles for bone tissue regeneration [179].

### 5.4. Cationic Liposomes

Cationic liposomes used as non-viral vectors are commonly composed of fatty acids linked to an alkyl functional group, which results in a positively charged head with a hydrophobic tail [180]. Felger reported the first use of cationic liposomes for a gene delivery therapy, through the successful embedding of pDNA into cationic liposomes and demonstration of their ability to compensate for the pDNA negative charge [181]. Felger demonstrated that this complex provided better interaction with the cell membrane and allowed for greater cellular uptake [181]. Since its discovery, cationic liposomes have been widely used in gene delivery because of their high affinity for the cell membrane and ease of production. One limitation of cationic liposomes is that they tend to bind to unspecific serum proteins, which can lead to their early removal by the phagocyte system [180]. Furthermore, while cationic lipids bearing ether bonds result in better transfection, they are non-biodegradable and lead to higher cytotoxicity. Conversely, cationic lipids including ester linkers are prone to biodegradation but are less cytotoxic [182].

Cationic liposomes have been used with greater success for the surface modification of calcium phosphate nanoparticles where they have effectively achieved nanoparticle stabilization. Zhou et al. achieved colloidal stability over a 21-day period and reported a ten-fold increase in transfection efficacy using lipid-functionalized CaP when compared to calcium phosphate alone [183]. Yang et al. investigated the surface functionalization of calcium phosphate using liposomes for siRNA delivery for cancer treatment [164]. They developed a simple lipid coating for a siRNA loaded calcium phosphate nanoparticle, which was then functionalized with PEG grafting to improve siRNA delivery. In order to further enhance siRNA delivery, they developed an asymmetric lipid functionalization approach. The calcium phosphate nanoparticles were initially coated with an anionic lipid, which entrapped the siRNA within the core, and then further coated with a cationic lipid which was PEGylated to shield charge effect and linked to an anisamide ligand [164]. Yang et al. reported a 40-fold improvement in transfection efficacy in vitro and a 4-fold increase in vivo, when using this lipid-calcium phosphate vehicle compared to a lipid-protamine one [164]. Other groups have used of 1,2-Dioleoyl-sn-glycero-3-phosphate (DOPA) lipid to functionalize aqueous-core calcium phosphate nanoparticles (Figure 3C) [157]. Schmidt et al. obtained DOPA-CaP particles of 120 to 185 ± 50 nm in size, with a shell thickness of 10 to 40 nm; the size growth was controlled by adding a carboxyethylphosphate capping molecule [157]. These nanoparticles were stable for over one month, even in the presence of proteinases and appeared to be suitable for delivery of hydrophilic cargoes [157].

### 5.5. Cell-Penetrating Peptides

Cell-penetrating peptides (CPP) are short sequences of amino acids, usually composed of 5–30 units, with the characteristic of easily penetrating the cell membrane while delivering a therapeutic cargo (e.g., RNAi). Penetration of the cellular membrane using a CPP is subject to controversy as the pathways by which it enters the cell is not entirely understood. Depending on the peptide structure, cargo and experimental conditions, different penetration mechanisms have been identified in the case of significant cargoes, e.g., clathrin-dependent [184] and clathrin-independent pathways [185], lipid rafts [186], and macropinocytosis [187]. CPPs are broadly divided into three classes: (1) cationic, (2) amphipathic and (3) hydrophobic CPPs. Cationic and amphipathic CPPs have been used to functionalize calcium phosphate nanoparticles for gene delivery applications [188].

Cationic-based CPPs represent the majority of CPPs and are characterised by their high positive charge and short amino acid sequences, and are considered very efficient in crossing the cell membrane [189]. The most common cationic CPPs are TAT peptide, penetratin and poly-arginine. Of these, only poly-arginine has been explored extensively for the functionalization of calcium phosphate nanoparticles. Arginine has a guanidium head group that can bind to the negatively charged cell membrane through hydrogen bonding, which leads to cell penetration at physiological pH [190]. Futaki et al. reported that for arginine CPP eight positive charges are the optimal configuration for efficient delivery [187]. Arginine-rich peptides, for example the Arg-Gly-Asp peptide sequence RGD, have been successfully used for calcium phosphate functionalization to improve siRNA delivery (Figure 3D). Bakan et al. reported the addition of arginine CPP did not alter the mean particle size of the calcium phosphate-siRNA nanoparticles [74]. However, it did change the zeta potential of the complex from negative to positive, thereby improving the interaction with the cellular barrier [74].

Amphipathic classified CPPs are chimeric amino acid sequences and are composed of both a hydrophilic and a hydrophobic group. Based on their structure, they can be distinguished in alpha-helical, beta-sheet structure, and proline-rich amphipathic CPP [189]. The pVEC [191,192], Pep-1 [193], and RGD peptides [194] are among the most common amphipathic CPPs, though they are not widely used in conjunction with CaP nanoparticles at present. Other examples of amphipathic CPPs have been synthesized for delivery of genetic cargoes. McCarthy et al. developed a novel peptide called RALA composed of sequences of arginine-alanine-leucine-alanine [195]. They combined the GALA and KALA peptides, where the lysine group in KALA has been substituted with arginine to overcome the limitation concerning cytotoxicity for lysine [195]. They further demonstrated how the improved α-helicity of RALA, which is pH-responsive once the peptide reaches the endosome, can represent a tool to facilitate nucleic cargo delivery [195]. Bennett et al. have obtained promising results for the delivery of RNAi using RALA peptide both in vitro and in vivo [196]. Sathy et al. subsequently exploited the use of RALA to enhance delivery of CaP nanoparticles, proving the positive effect of RALA-CaP in stimulating osteogenic markers and facilitating mineralization both in vitro and in vivo [165]. Overall, CPPs have shown to be very useful when used to functionalize inorganic nanoparticles, and much research has been conducted into their use in modifying gold nanoparticles and quantum dots, especially for cancer and imaging applications [190,197]. Research associated with the functionalization of calcium phosphate nanoparticles for effective RNAi delivery for bone tissue regeneration is still in its infancy. However, amino acid functionalization of calcium phosphate nanoparticles can be beneficial for enhanced direct delivery through the cell membrane, as the use of amino acids can provide multiple binding sites for RNAi, allowing the formation of more stable compounds [198].

## 6. Conclusions and Future Perspective

Recently, the application of gene therapy for bone tissue regeneration has attracted significant attention, offering the possibility to guide cellular fate without the administration of high quantities of drugs or protein. The use of calcium phosphate nanoparticles has been shown to facilitate the delivery of miRNAs and siRNAs, through the formation of stable complexes that can efficiently be endocytosed by targeted cells, allowing for the cytoplasmic release of miRNAs and siRNAs. This feature, in tandem with the natural biocompatibility of calcium phosphate and its promotion of bone mineralization, highlights the potential use of calcium phosphate nanoparticles as non-viral vectors for the treatment of a wide variety of bone diseases. However, reduced transfection efficacy of calcium phosphate nanoparticles remains a significant barrier to their use in clinical applications. For increased therapeutic response and transfection efficacy, calcium phosphate nanoparticles must demonstrate long-term stability under physiological conditions, reduced agglomerate formation, a mean particle size ≤200 nm and positive zeta potential for better interaction with the cell membrane. Surface functionalization of calcium phosphate nanoparticles offers the potential to overcome transfection limitations through surface modification of the nanoparticle to achieve the desired physical properties. The overall goal of these surface functionalization techniques is to maximize transfection efficiency by striking the optimal balance between protection of the genetic cargo and efficient release. Of the approaches discussed here PEG-ylation has been shown to offer improved CaP is more stable due to the colloidal stability of PEG, while generally cationic liposome coating methods result in an improved transfection efficiency due to the cationic characteristics of the lipids. Novel approaches have investigated the possibility of combining more than one functionalization method, e.g., an initial surface modification process to ensure calcium phosphate stabilization and then a further surface treatment method to improve intracellular endosomal escape. The majority of research has been directed towards the stabilization of calcium phosphate-RNAi nanoparticles for applications within the fields of cancer and bioimaging. However, the concepts proven for such applications also have potential for bone tissue regeneration applications, since both the bonding interactions between the calcium phosphate and RNAi and the molecular interactions between the functionalized calcium phosphate surface and the cell membrane would be similar. Specifically, the application of CPP-based systems to improve calcium phosphate transfection efficiency seems extremely promising-though the underlying mechanisms of the peptide-cell interaction are not yet well understood and require further investigation.

Finally, limited clinical translation of calcium phosphate-based gene delivery is primarily due to the lack of understanding of their behavior in vivo. As bone-related disease and bone injuries are generally not life-threatening conditions, risk-benefit concerns relating to the use of gene therapy in the treatment of these conditions requires consideration. A deeper understanding of the mechanistic principles of the in vivo bone formation process is required, especially concerning the role of miRNAs and their interplay during bone physiology. Furthermore, in order to design efficacious and clinically relevant therapies, a greater understanding of the role and target of each miRNA is required, along with identification of the optimal time frame for therapeutic delivery.

## Figures and Tables

**Table 1:** MicroRNA for regulation of bone markers and their effect on targeted genes during bone repair.

Cell Type	MicroRNA	Effect	Target	Reference
Osteoblast	miR-17-92	Promotes osteoblast differentiation	RUNX2, type I collagen	[27]
	miR-26a	Promotes osteoblast differentiation	RUNX2, VEGF, GSK-3β	[21,23,28]
	miR-29a	Unclear	RUNX2, type I collagen, type 5 collagen	[29,30]
	miR-29b	Promotes osteoblast differentiation	TGF-β, HDAC4	[30,31]
	miR-133a	Inhibits osteoblast differentiation	RUNX2	[32]
	antagomiR-133a	Promotes osteoblast differentiation	RUNX2	[33]
	miR-125b	Inhibits osteoblast differentiation	Unknown	[34]
	miR-135	Inhibits osteoblast differentiation	Smads5	[32]
	miR-138	Inhibits osteoblast differentiation	FAK	[35,36]
	miR-141	Inhibits osteoblast differentiation	DLX-5, Wnt signalling	[37,38]
	miR-196a	Promotes osteoblast differentiation	RUNX2, OPN	[39]
	miR-210	Promotes osteoblast differentiation	TGF-β, VEGF	[40]
	miR-335	Unclear	RUNX2, DDK1	[41,42]
	miR-338	Inhibits osteoblast differentiation	RUNX2, FGFR2	[43,44]
	miR-378	Promotes osteoblast differentiation	CASP3	[45,46,47]
	miR-637	Inhibits osteoblast differentiation	Osterix	[43,48]
	miR-2861	Promotes osteoblast differentiation	HDAC5	[49]
Osteoclast	miR-21	Promotes osteoclast differentiation	RANKL	[50]
	miR-29b	Unclear	Cdc42	[51]
	miR-31	Promotes osteoclast differentiation	RhoA pathway	[52]
	miR-34a	Inhibits osteoclast differentiation	Tgif2	[53]
	miR-155	Inhibits osteoclast differentiation	SOCS1	[54]
	miR-223	Unclear	Unclear	[55,56]
	miR-503	Promotes osteoclast differentiation	RANK	[57]
Chondrocytes	miR-140	Promotes chondrocyte differentiation	Dnpep	[58]
	miR-145	Inhibits chondrocyte differentiation	Sox9	[59]
	miR-199a*	Inhibits chondrocyte differentiation	Smad1, Smad4	[60]

**Table 2:** Advantages and limitations of calcium phosphate nanoparticle surface functionalisation methods.

Surface Functionalisation Method	Advantages	Limitations	Examples	Particle Size (Mean nm)	Transfection Efficiencies (%)
PEG-ylation	Improves calcium phosphate particle stability preventing particle growth Enhances particle protection allowing increased circulation time Improves biocompatibility Low immunogenicity Low cytotoxicity	Inhibits cellular uptake and endosomal escape	PEG-ALE coated CaP-siRNA [155] PAsp(DET)-PEG-siRNA-CaP [158] CaP/PEG-PAA/siRNA [159] PEG–SS–siRNA/CaP [160]	260 42 ± 5 100–300 90–120	Not provided ~82 ~95 76–86
Cationic polymers	Increases transfection efficacy Increases calcium phosphate stabilization	Mostly non-degradable Cytotoxic	siRNA-CaP-PEI [149] CaP-siRNA-tyrosine-grafted PEI (PEIY) [161]	316 ± 51 56–60	~70 ~95
Natural polymers	Excellent biocompatibility Biodegradable Low toxicity	Low transfection efficiency Poor stability after cellular uptake	CaP-siRNA-chitosan-glutamine [162] CaP/siRNA/DOPA-chitosan [156] CaP/siRNA/DOPA–hyaluronic acid [150] DOPA–hyaluronic acid/CaP/DNA/miRNA [163]	119 131.0 ± 2.1 63 to 278 ~50–250	91 ± 6 ~55 45–70 99.9
Cationic liposomes	Increases calcium phosphate stabilization	Cytotoxic Inflammatory response Early removal by the phagocyte system	Lipid calcium phosphate nanoparticles [164]	~40	~91
Cell-penetrating peptides (CPPs)	Enhances direct delivery through the cell membrane Increases calcium phosphate stabilization Increases transfection efficiency	Rapid degradation by enzymes within body fluids	Arginine CPP CaP-siRNA [74] Arginine-alanine-leucine-alanine (RALA)-CaP [165]	95–171 nm >100 nm	Not provided Not provided
